# Plant-Based Fat, Dietary Patterns Rich in Vegetable Fat and Gut Microbiota Modulation

**DOI:** 10.3389/fnut.2019.00157

**Published:** 2019-10-11

**Authors:** Jananee Muralidharan, Serena Galiè, Pablo Hernández-Alonso, Monica Bulló, Jordi Salas-Salvadó

**Affiliations:** ^1^Human Nutrition Unit, Department of Biochemistry and Biotechnology, Universitat Rovira i Virgili (URV), Reus, Spain; ^2^Institut d'Investigació Sanitària Pere Virgili (IISPV), Reus, Spain; ^3^Consorcio CIBER, M.P., Physiopathology of Obesity and Nutrition (CIBEROBN), Instituto de Salud Carlos III (ISCIII), Madrid, Spain; ^4^Unidad de Gestión Clínica de Endocrinología y Nutrición del Hospital Virgen de la Victoria, Instituto de Investigación Biomédica de Málaga (IBIMA), Málaga, Spain

**Keywords:** gut microbiota, plant-based fats, nuts, vegetable oils, Mediterranean diet

## Abstract

Diet is advocated as a key factor influencing gut microbiota. Several studies have focused on the effect of different carbohydrates, mainly fiber, on gut microbiota. However, what remains to be elucidated is the impact of a key component of diet that is widely debated upon: dietary fats. This review highlights the importance of understanding the source, quality, and type of fats that could differentially modify the intestinal microbiome. Fats from plant-based sources such as nuts, or vegetable oils have shown positive alterations in gut microbiota biodiversity both in *in vivo* and *in vitro* studies. Nuts and other plant-based fat sources, dietary patterns (e.g., Mediterranean diet) rich in polyunsaturated and monounsaturated fats and, in some cases, polyphenols, and other phytochemicals, have been associated with increased bacterial diversity, as well beneficial butyrate-producing bacteria imparting a positive metabolic influence. It is with this interest, this narrative review brings together evidences on different plant-based fat sources, dietary patterns rich in vegetable fats, and associated changes in gut microbiota.

## Introduction

The significance of gut microbiota has grown from being just a hitchhiker to an active metabolic organ. The human gastrointestinal tract is composed of trillions of bacteria that play an important role in the host metabolism (1). This data directly suggest that the global microbiome potential is extremely high. Use of diet to alter gut microbiota as a potential therapeutic target is widely researched (2).

Dietary fibers are an important source for the fermentation of intestinal bacteria (3). An extensive amount of research has focused on understanding dietary fiber as a key part of plant-based diets (4, 5). However, other than dietary fibers, fractions of unabsorbed protein and dietary fat, reaches the large intestine and therefore can potentially be substrates that differentially influence the microbial system (6, 7).

Even though there are many studies in the context of high-fat diets and gut microbiota, studies differentiating them from plant and animal-based sources are relatively scarce. Irrespective of the type of fat, high fat diets (HFD) have frequently shown to induce an increase in the abundance of Firmicutes in comparison to the low fat diet (LFD) (8, 9). Linoleic acid, mainly coming from plant sources, are utilized by different gut microbial species to produce conjugated linoleic acid (CLA) that has shown anti-inflammatory, anti-adipogenic, anti-diabetogenic, and anti-carcinogenic properties (10). Omega-3 fatty acids [ω-3 polyunsaturated fatty acids (PUFAs)] have received higher attention from scientific community due to its protective effects against inflammatory status both in *in vitro* and *in vivo* studies, compared to other types of fat (11), but its effects on microbiota regulation remain unclear [reviewed in (12)]. Unlike ω-3 PUFAs, monounsaturated fatty acids (MUFAs) have shown inconsistent results. In fact, a recent systematic review has shown that diets high in MUFA tend to decrease total bacterial numbers (13). Western diets rich in saturated fats and low in antioxidants, phytosterols, and other phytochemicals have shown to change gut microbiota favoring a pro-inflammatory state (14). Based on long-term dietary habits, gut microbial profile is divided broadly into two enterotypes: (i) *Prevotella* enterotype, found predominantly in the people consuming carbohydrate-based diets or the vegetarian diet; (ii) *Bacteroides* enterotype, found in high protein and/or animal products-derived diets (15).

With existing research, a diet with emphasis on plant-based foods and low consumption of red meat has been endorsed as a healthy dietary choice. Vegetarian or vegan diets (16–18) and the Mediterranean Diet (MedDiet) emphasizing the consumption of plant-based foods have shown to have beneficial impacts on gut microbiota (19), overall metabolism and health (20). Amongst these diets, MedDiet contains a high amount of plant-based fats (35–45% of total energy), sourced from olive oil [mainly extra virgin olive oil (EVOO)] and nuts. High-fat energy dense foods such as nuts or olive oil could be seen as foods contributing to weight gain that could lead to obesity or related morbidities. However, nuts and olive oil have not been associated with weight gain (21, 22), rather a direct association of these fat sources with healthy metabolic profiles has been shown (23), mainly ascribed to their specific fat composition and their bioactive molecule content.

### Animal vs. Plant Fat

Prior animal studies have shown that the composition, and not the quantity of dietary fat, is important in modulating endotoxemia (24). Circulating endotoxins, majorly from the gram-negative bacteria, elicit inflammation. Serum endotoxins from human and animal studies depict that after ω-3 PUFA intake, the post-prandial serum endotoxin production is lower than that of saturated fatty acids (SFAs) (24, 25). The majority of these studies have considered the SFA source from vegetables (butter or palm oil), and fish oil as the major ω-3 PUFA source. As plant-based fats vary widely by composition, future studies comparing different plant-based fat sources will be of profound value. Interestingly, animal foods such as red meat and fish are not only sources of fats, but also sources of protein. In a study focusing on different protein sources, it was noted that soy-based protein had the highest circulating endotoxins compared to red or white meat sources (26). Even though plant protein in this study showed higher endotoxin levels, evaluation of animal sources should be considered skeptically due to the presence of heme, N-nitroso compounds, polycyclic aromatic hydrocarbons and heterocyclic amines in meat products that are involved in gut health-related problems (27).

With the growing popularity of vegetarianism, many studies have investigated the differences in gut microbiota with respect to plant-based diets (vegan or vegetarian) (5, 28, 29). Considering the wide range of fat sources available, only few studies have explored their effects on gut microbiota. The complex nature of food makes it difficult to determine the causal nature of a particular dietary component on gut homeostasis. Hence, when the synergistic effects of a food are considered, plant-based fat sources also rich in antioxidants and fibers would be a better substitute to animal-based fat sources carrying heme and/or nitroso-compounds.

Even though this is a growing area of research, the collection of literature in bringing together evidences keenly on the different fat sources from plant-based diets and their effects on gut microbiota is limited. Hence, the purpose of this narrative review is to summarize the relevant evidence (after reviewing in PubMed) linking the different plant-based fat sources and dietary patterns rich in vegetable fat sources and their impact on gut microbiota. We selected the articles by using a combination of search terms in PubMed for each section. The following keywords were included in each section: (i) nuts, pistachios, hazelnuts, cashews, walnuts, macadamia nuts, peanuts, almonds, brazil nuts, pine nuts, pecans, (ii) corn oil, castor oil, coconut oil, cottonseed oil, sunflower oil, olive oil, rapeseed oil, peanut oil, palm oil, rice bran oil, safflower oil, sesame oil, soybean oil, plant-based fat, (iii) Mediterranean diet. All the above-mentioned keywords were used in combination with an “AND” builder with the following keywords: gut microbiome, gut microbiota, intestinal microbiome. We included only human studies or those conducted on mice or rats. *In vitro* studies were included only in the appropriate places where there was not enough evidence from human or mouse/rat studies. Despite that, we cannot discard that some studies may not be included as this is not a systematic review.

## Nuts and Gut Microbiota

Consumption of nuts has been shown to have protective effects against metabolic disorders such as type 2 diabetes (T2D), dyslipidemia, and cardiovascular disease (CVD). A recent prospective analysis conducted with 16,217 subjects with T2D showed that participants consuming ≥5 servings of nuts compared to ≤1 serving per month had a lower total CVD incidence, coronary heart disease incidence, CVD mortality and all-cause mortality (30). Previous meta-analysis reported a reduced risk for T2D, neurodegenerative disease, infectious diseases, with consumption of 28 g of nuts/day. Modulation of lipid metabolism, antioxidant activity and gut microbiota are some of the proposed mechanisms (31). Some of these benefits are driven by modulation in lipid metabolism, antioxidant activity, also via gut microbiota.

Nuts are a complex matrix of nutrients especially rich in fiber, unsaturated fatty acids (UNFAs) and different bioactive compounds such as tocopherols, phytosterols, phenolic compounds, and minerals such as magnesium (32). Some of these nutrients can reach the colon intact, being able to change the gastrointestinal microbiota composition and function. Different nutrients and their metabolites, such as polyphenols have shown to aid in gut microbiota balance and growth of beneficial bacteria [reviewed in (33)]. The fermentation of fiber from nuts or other sources to beneficial end-products (e.g., butyric acid) and the biotransformation of phytochemicals have been reported to be associated with the transition to a healthier microbiota (34). Thus, nuts could exhibit prebiotic effects by enriching potentially beneficial microorganisms such as Bifidobacteria or lactic acid bacteria (35).

Fat from nuts may have also a major impact on gut microbiota because a considerable amount of fat present in nuts can arrive intact to the colon. Incomplete mastication or inaccessible fats inside cell structures remain unabsorbed during digestion and this small degree of fat moves to the intestine, serving as a prebiotic (36, 37). Atwater factors of almonds (38), pistachios (39), walnuts (40), and cashews (41) have indeed showed an overestimation of measured energy contents.

Among nuts, almonds, pistachios, and walnuts have showed to have different protective properties modulating, for example, insulin resistance, glucose metabolism, and lipid profile [reviewed in (42), (43), and (44)]. However, their prebiotic properties were not well-characterized until a few years ago. Different *in vitro* and *in vivo* studies have analyzed the prebiotic effect and fermentation properties of raw and roasted almonds, as well as almond skins. These studies have shown the ability of different components of almonds that could positively alter the composition of gut bacteria (45–47). In fact, a stimulatory effect on *Lactobacillus* spp., and *Bifidobacterium* spp., has been observed from raw and roasted almond consumption (47). Beyond almonds, several clinical feeding trials have demonstrated a modulatory effect of other types of nuts on gut microbiota. First in 2014, Ukhanova et al., performed two separated randomized, controlled, cross-over feeding studies with healthy subjects, giving them either almonds (*n* = 18) or pistachios (*n* = 16), in three interventions (no nuts, 42 or 84 g/day) each for 18 days (48). They showed that both types of nuts significantly affected microbiota. However, the prebiotic effect of pistachio intake on gut microbiota composition was much stronger than that of almond consumption. Moreover, pistachios increased the number of butyrate-producing bacteria, identified as potentially beneficial, whereas the numbers of *Bifidobacterium* were not affected by the consumption of either type of nut (48). Relevantly, a 4-month, crossover randomized clinical trial (RCT) conducted in 49 pre-diabetic subjects found a shift toward a healthier gut microbiota following pistachio consumption by assessing gut-derived metabolites in 24 h-urine (49). Three metabolites related with gut microbiota metabolism (i.e., hippurate, p-cresol sulfate and dimethylamine) decreased after pistachio diet compared with the nut-free control intervention.

In 2014, Liu et al., reported a 6-week study with 48 volunteers that were randomly assigned to three different intervention groups: (i) control group was supplied with 8 g/d of fructooligosaccharides; (ii) intervention group supplemented with 10 g/d of almond skins; and, (iii) intervention group with 56 g/d of roasted, unsalted, whole almonds (50). *Bifidobacterium* spp., and *Lactobacillus* spp., increased significantly in the almond and almond skin groups. The populations of *Escherichia coli* mildly changed, and the growth of *Clostridium perfringens* was significantly repressed in both almond intervention groups. The difference in the results of these two studies could be attributed to their duration, since Ukhanova et al. (48) administered nuts only for 18 days in contrast with 6 weeks in the case of Liu et al. (50). Another 3-week short-term nut crossover study was conducted in 29 parents and their respective children (*n* = 29). The parent-children duo consumed 42 and 14 g/d of almonds (including almond butter), respectively. Researchers reported significant changes at overall genus level after almond consumption vs. control intervention, especially in children (51).

A controlled-feeding randomized crossover study conducted in 18 healthy subjects assessed the beneficial effect of almond consumption on gut microbiota composition for periods of 3 weeks (52). This study compared the effect of consuming 1.5 servings of raw or processed (roasted or chopped) almonds or almond butter to a control almond-free intervention group. They showed that almond consumption increased the relative abundances of *Lachnospira, Roseburia*, and *Dialister*. Particularly, chopped almonds increased the abundance of *Lachnospira, Roseburia*, and *Oscillospira*, while whole almonds increased *Dialister*, compared to control. Overall, this study showed that almond consumption and its degree of processing differentially impact the relative abundances of bacteria genera in the gastrointestinal tract.

Two different trials were recently performed to assess the shift in the gut microbiota due to walnut consumption with a different length of intervention (53, 54). Holscher et al., evaluated using a 3 weeks crossover study design (1 week washout) the effect of 42 g of walnuts vs. no consumption, in 18 overweight but otherwise healthy men and women (53). Forty-nine to sixty percent higher relative abundance of *Faecalibacterium, Clostridium, Dialister*, and *Roseburia* and 16–38% lower relative abundances of *Ruminococcus, Dorea, Oscillospira*, and *Bifidobacterium* were observed in walnut consumption compared to the control period. Moreover, authors reported an improvement in the lipid profile in case of walnut supplementation. These results are supported by *in vivo* studies indicating that walnuts increased the relative abundances of *Firmicutes*, including the genera *Clostridium* (55) and *Roseburia* (56). In fact, walnuts showed mild protection to the colon against a potent carcinogenic reaction partially due to walnut-induced changes to the gut microbiome (55). Due to the negative association of *Faecalibacterium* and *Roseburia*, positive association of *Oscillospira* with age, it has been suggested that consumption of walnuts may help in age related changes in the gut microbiota (57, 58). Future studies assessing the aspects of walnut consumption on gut microbiota and aging would be of value.

In a similar—but of a longer duration—crossover RCT, 135 normo-weight or overweight healthy subjects consumed 43 g/d of walnuts or a nut-free diet for 8 weeks (54). Generalized UniFrac distance showed that walnut consumption significantly changed microbiome composition and diversity. By using multidimensional scaling approach, authors reported dissimilarities of ~5% between walnut and control diet interventions. Specifically, the abundance of the family *Ruminococcaceae* and genus *Bifidobacterium* increased significantly, while the genus *Blautia* and *Anaerostipes* decreased significantly during walnut consumption. A controlled feeding intervention study with roasted hazelnuts was conducted in hyperlipidemic (and age-matched normolipidemic) children and adolescents (7–17 years) for 8-weeks assessing the changes in gut microbiota. At baseline, the α- and β-diversity microbiota were significantly different between hyperlipidimic and normolipidemic participants. At baseline, subjects with hyperlipidemia had significantly lower concentrations of acetate, butyrate and propionate, whereas they had significantly higher levels of lactate, pyruvate and isobutyrate. The authors reported a non-significant difference in the microbial composition after the hazelnut intervention between the hyperlipidimic and control participants. In SCFAs' (measured in feces), only a significant increase in acetate concentrations was reported after the intervention in the hyperlipidimic group (59).

Taken together, although daily consumption of nuts (1–2 servings/d) have shown to impact gut microbiome by enhancing beneficial bacterial species, further studies are needed to determine whether: (i) these modulations are preserved during longer nut consumption periods; (ii) these modulations may also affect subjects with cardiometabolic diseases; and (iii) these modulations are associated with improvements in other disease-related parameters.

## Vegetable Oils and Gut Microbiota

A common and popular plant-based fat source is vegetable oil. Consumption of vegetable oils rich in unsaturated fats has been associated with healthier metabolic conditions (low LDL (low density lipoprotein) cholesterol levels, and lower risk of T2D and CVD compared to other animal fat sources) (60, 61). This could be partly attributed to the type of fat but also to their high content in polyphenols and other phytochemicals in case of virgin olive oil (62). Vegetable oils are formed by a mixture of SFAs, UNFAs, MUFAs, or omega-6 polyunsaturated fatty acids (ω6 PUFAs), which can vary between different types of oils. Even though vegetable ω6 PUFAs have been considered pro-inflammatory in contrast to ω3 fatty acids, the interaction of omega-3 and omega-6 fatty acids in the context of inflammation is complex and still not properly understood (63–65).

Avocados are an important plant-based fat source that are also rich in dietary fibers. Only few studies have been conducted to explore the effects of avocado on gut microbiota. A recent RCT conducted amongst 160 adults (BMI ≥ 25 Kg/m^2^) with parallel arms of treatment (iso-caloric meals, with or without avocado), evaluated the effect of Hass avocado consumption for 12 weeks. Compared to control, avocado consumption increased acetate (*p* < 0.01) and total SCFA's (*p* = 0.02) and the relative abundances of *Faecalibacterium* (*p* = 0.01) in feces (66). In a similar RCT with 51 healthy overweight/obese participants, the effect of avocado consumption on gut microbiota, biomarkers of inflammation, weight loss and body composition was tested. Participants either followed an avocado hypocaloric diet (1 Hass avocado- AVO) or a hypocaloric diet avoiding the consumption of avocados (CTRL) for 12 weeks. Relative proportions of genus *Bacteroides, Clostridium, Methanospaera*, and *Candidatus Soleaferrea* were altered significantly in the AVO group compared to CTRL group. Also a trend to decrease serum inflammatory markers IL-1β (*P* = 0.07) and C-reactive protein (*P* = 0.074) was observed in the AVO group compared with CTRL group (67).

Health benefits of olive oil, which is rich in MUFAs and polyphenols, has been largely related to a decrease in the incidence of CVDs and hypertension as well as being considered as a positive modulator in cognitive functions (68, 69). Olive oil can be categorized into four types based on the processing methods and its contents: extra-virgin olive oil, virgin olive oil, refined olive oil (ROO), and Orujo oil (68). Even though the main fatty acid composition remains the same, some polyphenolic components change in these four types of olive oil. Virgin olive oil has the highest polyphenol content (~150–400 mg/kg), refined olive oil with the lowest polyphenol content (~0–5 mg/Kg), and the common olive oil, pomace olive oil with intermediate polyphenol content (~10–100 and ~10–30 mg/Kg, respectively) (70). It is important to understand the difference in properties exerted by polyphenols in comparison to the fat profile of olive oils. With this regard, Hidalgo et al. (71) compared 12 week feeding of EVOO, ROO butter, and the standard chow diet in mice. Denaturing gradient gel electrophoresis (DGGE) and culture-dependent methods were used to analyze the microbiota in the feces. The family *Lactobacillaceae* appeared to increase in the butter group from baseline to week 12. Most of the species reported in all the diet groups were uncultured and no quantitative statistical evaluation was performed comparing the differences in microbiota composition. Hence, it is difficult to state specific differences among diets and/or time points. It was noted that most EVOO microbiota clustered with ROO, while microbiota cluster from butter was different. Also, butter diet induced changes closer to the gut microbiota of obese individuals, whereas the EVOO in the opposite direction and ROO with an intermediate behavior (71). Hence, it was observed that even though polyphenol content of the olive oil contributes to an extent to the changes in gut microbiota, the fat profiles also play a determining role.

Prieto et al. (72) compared the effects of a diet enriched in EVOO vs. butter (BT) in 26 Swiss Webster mice. They were fed with a standard diet (SD, *n* = 8) (3% of total energy from fats) or one of the two high fat isocaloric diets (35% of total energy from fats) enriched in EVOO (*n* = 9) or butter (BT, *n* = 9). Mice fed with BT diet, showed the highest systolic blood pressure (SBP), and SBP was positively correlated with *Desulfovibrio*. EVOO group had the lowest plasma insulin, which was correlated inversely with *Desulfovibrio*. Several other correlations were observed between the gut microbiota (at phylum, family, genus and species levels) and the measured metabolic syndrome (MetS) parameters. The authors concluded a positive metabolic impact of EVOO mediated by the gut microbiota (72). Similar result with reduction in SBP was reported in another mice study fed with EVOO (73), in which the taxonomic cluster of Clostridia cluster XIVa was inversely correlated with SBP, and a significantly higher abundance of *Lactobacilli* was also seen in the EVOO group (73).

The quality of fats in terms of health is usually indicated by its levels of saturation or unsaturation. Recently a mice study was conducted to evaluate the differences amongst SFA, UNFAs on gut microbiota (8). Three different HFD (40% of total energy from olive oil, corn oil or milk fat) and a LFD were given to the mice for 12 weeks. This study not only evaluated the microbial changes in the gut, but also the host response to these changes, hence giving an overall picture on microbe-host homeostasis. All the HFD increased the abundance of Firmicutes. The following increased abundances were noted in each group: olive oil group (*Clostridiaceae, Peptostreptococcaceae, Ruminococcaceae*, and *Dorea* spp.); milk fat (*Erysipelotrichales* and several genera from *Ruminicoccus*); corn oil (*Turicibacteracea* and *Coprococcus* spp.). Acetic acid and propionic acid levels were decreased in the olive oil, corn oil group compared to the low-fat chow group, whereas milk fat had similar levels of SCFA to that of low fat chow group. Corn oil rich in ω6 PUFAs showed increase in risk factors for development of dysfunctional gut barrier, whereas the milk fat rich in SFA promoted host inflammation, and olive oil resulted in a less inflammatory environment compared to the other two diets (8).

Few studies have focused on the phenolic components of olive oil (74, 75) and their role in modulating gut microbiota. Phenolic compounds of olive oil in combination with thyme phenolic compounds have shown to increase in members of *Bifidobacterium* and decrease the oxidation of LDL in blood in hypercholesteremic participants (74). However, further research is required in elucidating the role of different components of olive oil on gut microbiota.

Flaxseed oil (FO), soybean oil, coconut oil, palm oil and canola oil are other types of vegetable fat sources that are usually consumed around the world. They vary from each other widely by fatty acids and bioactive components.

Palm oil and coconut oil are SFA rich vegetable oils. Comparing the vegetable fats based on their PUFA/SFA ratio, by supplementing either a HFD rich in palm oil, safflower oil or olive oil, had demonstrated that palm oil (having the lowest PUFA/SFA ratio) reduced the microbial diversity and increased the Firmicutes-to-Bacteroidetes ratio (9). Apart from palm oil, another vegetable fat rich in SFA source is coconut oil. Compared to palm oil, coconut oil is characterized by the presence of both medium- and long- chain fatty acids, which may have better implications for host energy balance than lipids rich in long-chain fatty acids. Coconut oil, in its virgin form (i.e., of higher quality) has shown to be associated with beneficial effects on secondary parameters of T2D in mice, along with an increased abundance of beneficial bacteria such as *Lactobacillus, Allobaculum*, and *Bifidobacterium* species (76). Recent results from animal studies comparing coconut oil vs. soybean oil based diets showed that soybean oil resulted in a detrimental metabolic health compared to coconut oil, however with no changes in cecal microbiota (77).

An interesting study compared gut microbiota composition after the consumption of either lard (rich in SFA), fish oil (rich in ω6-PUFA, MUFA) or soybean oil (rich in ω3-PUFA) as different source of fats in middle-aged rats (78). *In vitro* and *in vivo* studies showed a different gut microbiota structure in the fish oil group from soybean oil or lard groups. Fish oil group has the highest relative abundance of phylum Proteobacteria and genus *Desulfiovibrio*. Along with these observations, it was also noted that mRNA levels of inflammatory markers (IL-1β, IL-6, IL-17, IL-18, and TNF-α) were higher in the fish oil group. Both these results indicate that fish oil could potentially increase the risk of inflammation, contrary to the prior studies (79). In fact, a high content of PUFA in diet, even if recommended by public health, is considered to cause metabolic oxidative stress and inflammation (80), also high MUFA diet has been suggested to have less consistent effects on gut microbiota (13). Therefore, these results suggest a new insight into the potentially negative effect of fish oil on inflammation through changing the microbiota population, in contrast with a vegetable source of fats like soybean oil.

To better understand the role of different types of fats on metabolic health, four HFD enriched with either palm oil, olive oil, safflower oil, or a combination of both flaxseed oil and fish oil were fed to wild-type C57BL/6J male mice. The groups with high MUFA and PUFA contents (olive oil and flax plus fish oil) showed a lower plasma triglyceride and less weight gain. Also, different compositions in gut microbiota were found between groups. Especially, olive oil group was characterized by an increase in bacterial family of *Bacteroidaceae*, and flaxseed/fish oil group was the only one in which there was an increase in *Bifidobacteriacea* family. Both this bacterial family include commensal bacterial with beneficial effects on gut health (81).

Other than the soybean oil, FO is a plant-derived oil rich in ω3 PUFAs, mainly α-linolenic acid (ALA, 18:3 ω-3). Dietary FO has shown protection against acute alcoholic hepatic steatosis via ameliorating lipid homeostasis at adipose tissue-liver axis in mice (82). However, the impact of dietary FO on inflammation and gut microbiota in chronic alcoholic liver disease (ALD) remains unknown. In order to investigate this topic Zhang et al., evaluated the interplay among the diet, gut microbiota, inflammation and ALD in mice models of ALD (83). Sixty mice were randomly allocated into four groups: pair-fed (PF) with corn oil (CO) group (PF/CO); alcohol-fed (AF) with CO group (AF/CO); PF with FO group (PF/FO); AF with FO group (AF/FO). A reduction of *Porphyromonadaceae* and *Parasutterella*, and an increase in Firmicutes and *Parabacteroides*, were observed in AF group compared to the PF control. Supplementation of FO in the ethanol consumption group (AF/FO) reduced *Proteobacteria* and *Porphyromonadaceae* significantly compared with AF/CO group.

Canola Multicenter Intervention Trial (COMIT) evaluated the interactions between obesity status and dietary intake of mono- and poly-unsaturated oils on human gut microbiome with participants at MetS risk. The experimental diets used were: (1) conventional canola oil (Canola); (2) DHA-enriched high oleic canola oil (CanolaDHA); (3) high oleic canola oil (CanolaOleic); (4) blend of two PUFA-rich of corn/saffower oil (25:75, CornSaff); and (5) blend of flax/saffower oil (60:40, FlaxSaff) supplemented diets designed to maintain body weight during the treatment periods. Diets 1, 2, 3 were rich in MUFAs and diets 4, 5 rich in PUFAs. Clear differences were observed in the gut microbiota profiles of obese group vs. overweight and the normal weight participants, with Firmicutes dominating the obese group. The differences between MUFA and PUFA rich diets, continued to be segmented by the influence of BMI. Abundance of *Faecalibacterium* [which has shown anti-inflammatory properties (84)] differed across treatments, with highest abundance in CanolaOleic and lowest in CanolaDHA, indicating the potential of oleic acid with an anti-inflammatory property (85).

**Figure 1:**
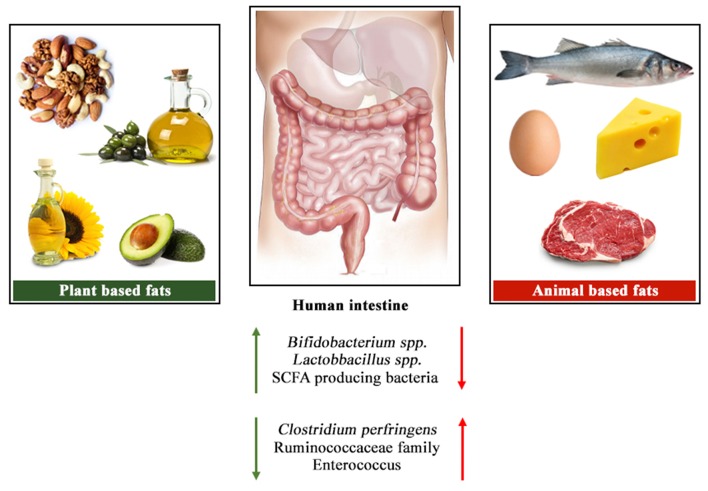
Overview of the changes in the gut microbial profile with differences in animal and plant-based fat sources.

Figure 1 shows an overview of the changes in the gut microbial profile with differences in animal and plant-based fat sources. Even though plant-based oils have been part of our diet since many decades, the potential impacts of these oils on gut microbiota still remain relatively unknown. The ratio of different saturated or unsaturated fatty acids clearly impose different effects on gut microbiota, however the debate remains open on the levels that is most suitable for better gut health.

## Mediterranean Diet and Gut Microbiota

The traditional view of single nutrient health effects has been shifting toward synergy of multiple food components and dietary patterns. The traditionall view of single nutrient health effects has been shifting toward synergy of multiple food components and dietary patterns. Understanding the effects of nutrient components and dietary patterns would be helpful to make lifestyle recommendations (86). Plant-based diets have been gaining acceptance and popularity due to the positive health benefits. Modulation of gut microbiota is one of the plausible mechanisms explaining these benefits. In terms of nutritional content, most of the plant-based diets are low in total and saturated fats compared to the omnivores diet (87, 88). However, MedDiet is an exception to this, with a high content of MUFA and PUFA from plant sources.

Several studies have emphasized the health effects of MedDiet since the seven countries study (89). MedDiet has been evaluated in terms of its effects on mortality, cardiovascular risks, mortality in several systematic reviews and meta-analysis (90, 91). The traditional MedDiet, is characterized by high consumption of vegetables, legumes, grains, fruits, nuts, and olive oil (plant-based foods), moderate consumption of fish and wine, and low consumption of red and processed meat and sugar. This dietary pattern rich in polyphenols, fiber and unsaturated fat, impart the above mentioned health benefits by various mechanisms including anti-oxidative potentials, anti-inflammatory properties and gut microbiota modulation (92) among others.

High-level adherence to MedDiet has shown to be positively associated with changes in beneficial gut microbiome and their metabolites (93). Contrary, a lower adherence to MedDiet was linked to higher urinary TMAO levels, a microbial metabolite that has been reported to be a marker for cardiovascular risk (94). Enhancement of fiber-degrading *Prevotella*, Firmicutes, and higher level of fecal short-chain fatty acids has been associated with higher adherence to a MedDiet (19). Similarly, presence of fiber degrading *Prevotella* was seen higher in preadolescent Egyptian subjects (*n* = 28) following a MedDiet in comparison to preadolescents in Dayton, USA (*n* = 14) consuming a Western diet (95). An observational study conducted in Greece amongst 120 participants investigated the associations between adherence to MedDiet and gut microbiota pattern. In this study, a higher adherence to MedDiet was inversely associated with *E. coli* counts, higher *Bifidobacteria: E. coli* ratio. Within the SCFA's measured, acetate was present in highest proportions (i.e., higher molar ratio) in all the tertiles of MedDiet adherence score (low, medium, and high). Also, greater molar ratio of acetate was reported to be significantly associated with higher adherence to MedDiet (96). MedDiet score measured in another study as an indicator of adherence to diet showed similar results (97). It was observed that the higher MedDiet score was associated with abundance of phylum Bacteroidetes, family *Prevotellaceae* and genus *Prevotella*. Fecal propionate and butyrate were higher in participants with a higher MedDiet score. Also, the consumption of olive oil, the main source of MUFA of this diet, was associated with increasing proportions of taxa *Tenericutes* and *Dorea* (97). In another study conducted in the Mediterranean population, genus *Dorea* and *Lactobacillus* were over represented in those participants consuming a high PUFA/SFA ratio (93). Haro et al. (98) conducted an intervention study comparing MedDiet (35% fat: 22% monounsaturated; 6% polyunsaturated and 7% saturated) and a low fat high complex carbohydrates (LFHCC) diet (28% fat: 12% monounsaturated; 8% polyunsaturated and 8% saturated) for a period of 1 year amongst 20 obese men. Consumption of MedDiet showed an increase in beneficial *Roseburia* genus whereas consumption of LFHCC showed an increase in fiber degrading *Prevotella* and *F. prausnitzii* (98). Another interesting study compared the MedDiet and a vegan diet (*Ma-Pi 2*). The *Ma-Pi 2* diet is rich in seaweeds, wholegrains, legumes and fermented products. Both diets followed for 3 days in 12 reactive hypoglycemic participants, induced no changes in the gut microbial composition, however the SCFA's in the *Ma-Pi 2* diet group was increased significantly from baseline to the 4th day (99).

Compromised gut bacterial profile is observed amongst people with several metabolic disorders (100, 101) Exploring MedDiet as a nutritional therapy could help in the reestablishment of a beneficial gut ecosystem. In this regard, few studies have evaluated the effect of MedDiet on gut microbiota and health. A total of 239 participants (with and without MetS) from the CORDIOPREV study were randomly allocated in two groups: LFD (MetS, *n* = 139) and MedDiet group (MetS, *n* = 101). After 2 years of following the diets, participants in the MedDiet group showed a restoration of some species of gut microbiota (*P. distasonis, B. thetaiotaomicron, F. prausnitzii, B. adolescentis*, and *B. longum*) in only those with MetS (102). MedDiet has also been effective in betterment of gut microbial ecology amongst Crohn's disease patients by increasing the Bacteroidetes phylum and *Clostridium* genus after 6 weeks of MedDiet consumption (103).

Even though overall credits on the beneficial effects of MedDiet cannot only be given to the healthy fat profile, it cannot be discarded that other components of this dietary pattern (such us dietary fiber, some vitamins and minerals, polyphenols and other phytochemicals) may also exert effects on gut microbiota profile and activity. Therefore, future larger human intervention studies are required in order to understand the role of MedDiet and its components on gut microbiota alterations.

## Discussions

Increasing number of studies are focusing on the importance of plant-based diets, as well as on the components of this type of diet. Nuts, olive oil and other plant fat sources comes with a broad composition of fatty acids that has varied biological impacts. Investigating the potential role of PUFAs in inducing beneficial effects should be evaluated with care, as the enzymatic peroxidation products of PUFAs has shown carcinogenic potentials (104). Studies exploring the cumulative effects of the fat source (containing other non-fat components such fiber or antioxidants) could mask the isolated effects of oxidation products of PUFAs (105, 106). These studies could strengthen the importance in understanding the mechanism involved in the synergy of different dietary components and fat on gut microbiota.

Exploration of novel pathways such as for sterculic acid that has shown effects on insulin resistance and obesity via gut microbiota modulation could be of interest to develop nutritional therapies (107). A comprehensive systematic review conducted by Wolters et al., observed that a modulation of dietary fat—by quantity or quality—did not impose any effects on gut microbiota in interventional studies, whereas observational studies reported gut microbiota shifts (13). A key reason discussed by the authors of these studies was the low intervention follow-up time. Moreover, gut microbiota studies are subjected to inter-individual differences that complicates the analysis. Further interventional studies with dietary fats, focusing on the aspect of gut microbiota would aid in a better understanding and to establish nutritional recommendations.

Dietary fat is an essential component of diet that needs to be consumed in the right quantity and quality. Based on the studies included in this review, nuts, and other plant-based fats seem to exert a favorable effect on genus *Bifidobacterium, Roseburia*, and *Faecilibacterium*, which has been associated with positive health effects. High fat diets with SFA as the main fat component have consistently been correlated with negative modulation of gut microbiota such as decreasing relative diversity. Thus, replacement of SFAs with plant sources of PUFAs and MUFAs, especially those rich in polyphenol and other phytochemicals, would help in positive modulation of gut microbiota and the corresponding health implications.

## Author Contributions

JS-S and MB contributed to the design of the review and editing of the manuscript. JM, PH-A, SG, MB, and JS-S performed the bibliographical search and wrote the first draft. All the authors approved the final manuscript.

### Conflict of Interest

JS-S reports serving on the board of the International Nut and Dried Fruit Council, the Danone International Institute, and the Eroski Foundation and receiving grant support from these entities through his institution. He also reports serving on the Executive Committee of the Instituto Danone Spain. He has also received research funding from the California Walnut Commission, Sacramento CA, USA; Patrimonio Comunal Olivarero, Spain; La Morella Nuts, Spain; and Borges S.A., Spain. He reports receiving consulting fees or travel expenses from Danone; the California Walnut Commission, the Eroski Foundation, the Instituto Danone - Spain, Nuts for Life and the Australian Nut Industry. The remaining authors declare that the research was conducted in the absence of any commercial or financial relationships that could be construed as a potential conflict of interest.
